# From outputs to insights: a survey of rationalization approaches for explainable text classification

**DOI:** 10.3389/frai.2024.1363531

**Published:** 2024-07-23

**Authors:** Erick Mendez Guzman, Viktor Schlegel, Riza Batista-Navarro

**Affiliations:** ^1^Department of Computer Science, The University of Manchester, Manchester, United Kingdom; ^2^ASUS Intelligent Cloud Services (AICS), ASUS, Singapore, Singapore

**Keywords:** Natural Language Processing, text classification, Explainable Artificial Intelligence, rationalization, language explanations

## Abstract

Deep learning models have achieved state-of-the-art performance for text classification in the last two decades. However, this has come at the expense of models becoming less understandable, limiting their application scope in high-stakes domains. The increased interest in explainability has resulted in many proposed forms of explanation. Nevertheless, recent studies have shown that *rationales*, or language explanations, are more intuitive and human-understandable, especially for non-technical stakeholders. This survey provides an overview of the progress the community has achieved thus far in rationalization approaches for text classification. We first describe and compare techniques for producing extractive and abstractive rationales. Next, we present various rationale-annotated data sets that facilitate the training and evaluation of rationalization models. Then, we detail proxy-based and human-grounded metrics to evaluate machine-generated rationales. Finally, we outline current challenges and encourage directions for future work.

## 1 Introduction

Text classification is one of the fundamental tasks in Natural Language Processing (NLP) with broad applications such as sentiment analysis and topic labeling, among many others (Aggarwal and Zhai, 2012; Vijayan et al., 2017). Over the past two decades, researchers have leveraged the power of deep neural networks to improve model accuracy for text classification (Kowsari et al., 2019; Otter et al., 2020). Nonetheless, the performance improvement has come at the cost of models becoming less understandable for developers, end-users, and other relevant stakeholders (Danilevsky et al., 2020). The opaqueness of these models has become a significant obstacle to their development and deployment in high-stake sectors such as the medical (Tjoa and Guan, 2020), legal (Bibal et al., 2021), and humanitarian domains (Mendez et al., 2022).

As a result, Explainable Artificial Intelligence (XAI) has emerged as a relevant research field aiming to develop methods and techniques that allow stakeholders to understand the inner workings and outcome of deep learning-based systems (Gunning et al., 2019; Arrieta et al., 2020). Several lines of evidence suggest that providing insights into text classifiers' inner workings might help to foster trust and confidence in these systems, detect potential biases or facilitate their debugging (Arrieta et al., 2020; Belle and Papantonis, 2021; Jacovi and Goldberg, 2021).

One of the most well-known methods for explaining the outcome of a text classifier is to build reliable associations between the input text and output labels and determine how much each element (e.g., word or token) contributes toward the final prediction (Hartmann and Sonntag, 2022; Atanasova et al., 2024). Under this approach, methods can be divided into feature importance score-based explanations (Simonyan et al., 2014; Sundararajan et al., 2017), perturbation-based explanations (Zeiler and Fergus, 2014; Chen et al., 2020), explanations by simplification (Ribeiro et al., 2016b) or language explanations (Lei et al., 2016; Liu et al., 2019a). It is important to note that the categories cited above are not mutually exclusive, and explainability methods can combine several. This is exemplified in the work undertaken by Ribeiro et al. (2016a), who developed the Local Interpretable Model-Agnostic Explanations method (LIME) combining perturbation-based and explanations by simplification.

Rationalization methods attempt to explain the outcome of a model by providing a natural language explanation (*rationale*; Lei et al., 2016). It has previously been observed that rationales are more straightforward to understand and easier to use since they are verbalized in human-comprehensible natural language (DeYoung et al., 2020; Wang and Dou, 2022). It has been shown that for text classification, annotators look for language cues within a text to support their labeling decisions at a class level (*human rationales*; Chang et al., 2019; Strout et al., 2019; Jain et al., 2020).

**Figure 1:**
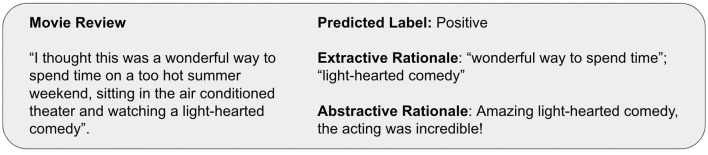
Example of an extractive and abstractive rationale supporting the sentiment classification for a movie review.

Rationales for explainable text classification can be categorized into *extractive* and *abstractive rationales* (Figure 1). On the one hand, extractive rationales are a subset of the input text that support a model's prediction (Lei et al., 2016; DeYoung et al., 2020). On the other hand, abstractive rationales are texts in natural language that are not constrained to be grounded in the input text. Like extractive rationales, they contain information about why an instance is assigned a specific label (Camburu et al., 2018; Liu et al., 2019a).

This survey refers to approaches where human rationales are not provided during training, as *unsupervised rationalization methods* (Lei et al., 2016; Yu et al., 2019). In contrast, we refer to those for producing rationales where human rationales are available as additional supervision signal during training, as *supervised rationalization methods* (Bao et al., 2018; DeYoung et al., 2020; Arous et al., 2021).

Even though XAI is a relatively new research field, several studies have begun to survey explainability methods for NLP. Drawing on an extensive range of sources, Danilevsky et al. (2020) and Zini and Awad (2022) provided a comprehensive review of terminology and fundamental concepts relevant to XAI for different NLP tasks without going into the technical details of any existing method or taking into account peculiarities associated with text classification. As noted by Atanasova et al. (2024), many explainability techniques are available for text classification. Their survey contributed to the literature by delineating a list of explainability methods used for text classification. Nonetheless, the study did not include rationalization methods and language explanations.

More recently, attention has been focussed on rationalization as a more accessible explainability technique in NLP. Wang and Dou (2022) and Gurrapu et al. (2023) discussed literature around rationalization across various NLP tasks, including challenges and research opportunities in the field. Their work, provides a high-level analysis suitable for a non-technical audience. Similarly, Hartmann and Sonntag (2022) provided a brief overview of methods for learning from human rationales beyond supervised rationalization architectures aiming to inform decision-making for specific use cases. Finally, Wiegreffe and Marasović (2021) identified a list of human-annotated data sets with textual explanations and compared the strengths and shortcomings of existing data collection methodologies. However, it is beyond the scope of this study to examine how these data sets can be used in different rationalization approaches. To the best of our knowledge, no research has been undertaken to survey rationalization methods for text classification.

This survey paper does not attempt to survey all available explainability techniques for text classification comprehensively. Instead, we will compare and contrast state-of-the-art rationalization techniques and their evaluation metrics, providing an easy-to-digest entry point for new researchers in the field. In summary, the objectives of this survey are to:

1. Study and compare different rationalization methods;
2. Compile a list of rationale-annotated data sets for text classification;
3. Describe evaluation metrics for assessing the quality of machine-generated rationales; and
4. Identify knowledge gaps that exist in generating and evaluating rationales.

From January 2007 to December 2023, our survey paper's articles were retrieved from Google Scholar using the keywords “rationales,” “natural language explanations,” and “rationalization.” We have included 88 peer-reviewed publications on NLP and text classification from journals, books, and conference proceedings from venues such as ACL, EMNLP, LREC, COLING, NAACL, AAAI, and NeurIPS.

**Figure 2:**
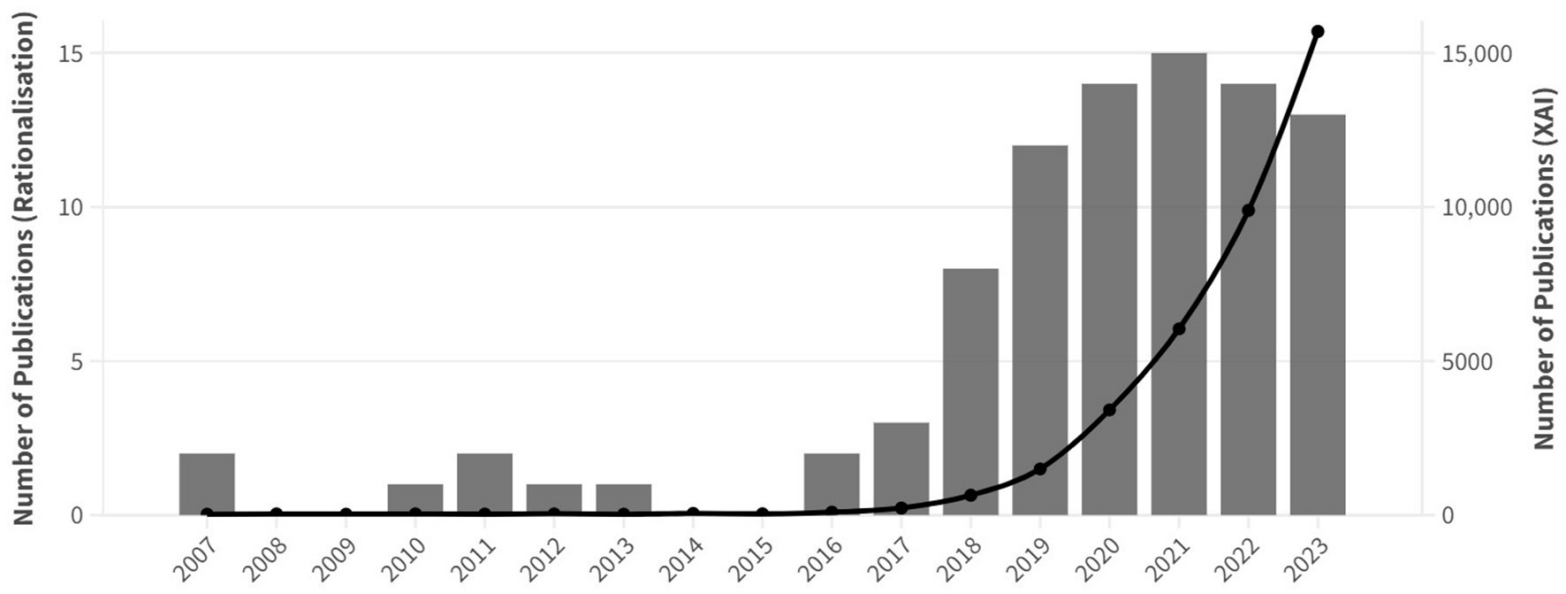
Evolution of the number of peer-reviewed publications on rationalization for text classification (bar chart, left y-axis) and XAI (line chart, right y-axis) from 2007 to 2023.

Figure 2 reveals that there has been a shared increase in the number of research articles on rationalization for explainable text classification since the publication of the first rationalization approach by Lei et al. (2016). Similarly, the number of research articles on XAI has doubled yearly since 2016. While the number of articles on rationalization peaked in 2021 and has slightly dropped since then to reach 13 articles in 2023, the number of publications on XAI has kept growing steadily. It is important to note that articles published before 2016 focus on presenting rationale-annotated datasets linked to *learning with rationales* research instead of rationalization approaches within the XAI field.

This survey article is organized as follows: Section 2 describes extractive and abstractive rationalization approaches. Section 3 compiles a list of rationale-annotated data sets for text classification. Section 4 outlines evaluation metrics proposed to evaluate and compare rationalization methods. Finally, Section 5 discusses challenges, points out gaps and presents recommendations for future research on rationalization for explainable text classification.

## 2 Rationalization methods for text classification

We now formalize extractive and abstractive rationalization approaches and compare them in the context of text classification. We define a standard text classification in which we are given an input sequence *x* = [*x*_1_, *x*_2_, *x*_3_, …, *x*_*l*_], where *x*_*i*_ is the *i*-th word of the sequence, and *l* is the sequence length. The learning problem is to assign the input sequence *x* to one or multiple labels in *y*∈{1, …, *c*}, where *c* is the number of classes.

**Figure 3:**
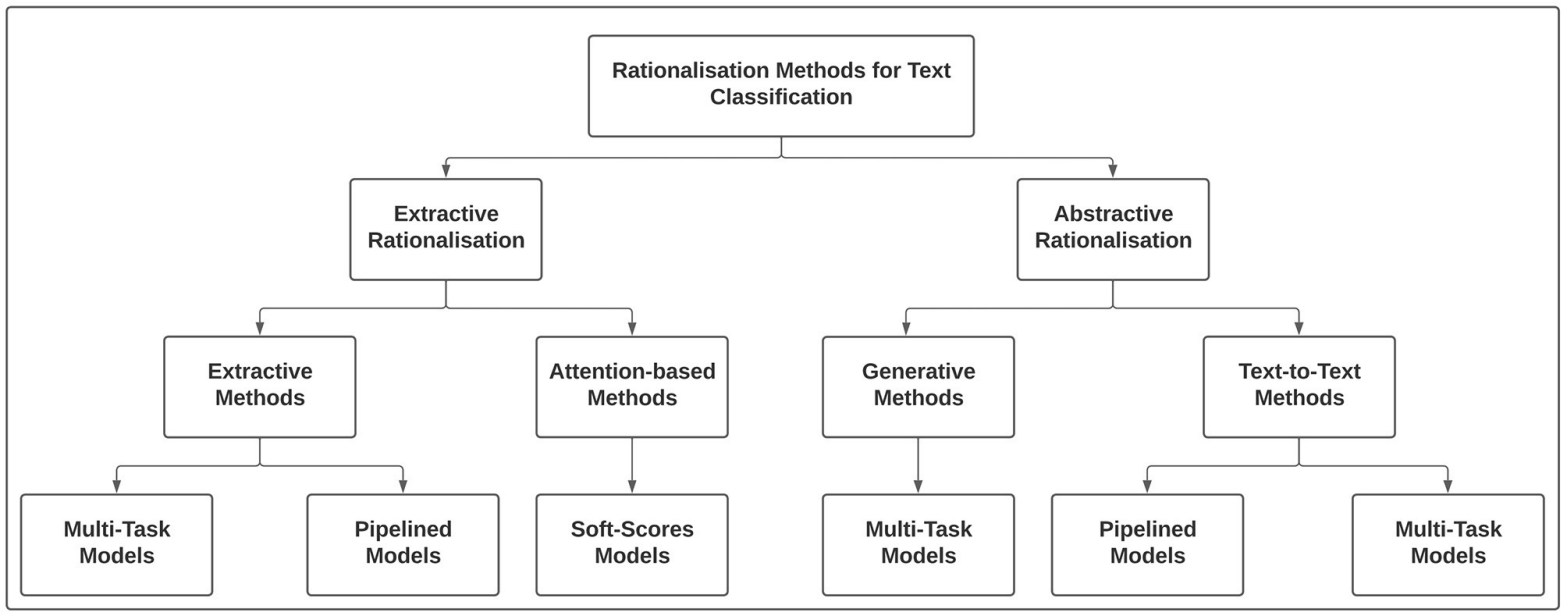
Overview of extractive and abstractive rationalization approaches in explainable text classification.

Figure 3 presents an overview of rationalization methods for producing extractive and abstractive rationales. While extractive rationalization models can be categorized into extractive or attention-based methods, abstractive rationalization models can be classified into generative and text-to-text methods. Finally, the component of both extractive and abstractive methods can be trained either using multi-task learning or independently as pipelined architecture.

### 2.1 Extractive rationalization

In extractive rationalization, the goal is to make a text classifier explainable by uncovering parts of the input sequence that the prediction relies on the most (Lei et al., 2016). To date, researchers have proposed two approaches for extractive rationalization for explainable text classification: (i) *extractive* methods, which first extract evidence from the original text and then make a prediction solely based on the extracted evidence (Lei et al., 2016; Jain et al., 2020; Arous et al., 2021), and (ii) *attention-based* methods, which leverage the self-attention mechanism to show the importance of words through their attention weights (Bao et al., 2018; Vashishth et al., 2019; Wiegreffe and Pinter, 2019).

Table 1 presents an overview of the current techniques for extractive rationalization, where we specify methods, learning approaches taken and their most influential references.

**Table 1:** Overview of common approaches for extractive rationalization.

**Approach**	**Method**	**Supervised**	**Representative paper(s)**
Extractive	Multi-Task	✗	Lei et al., 2016; Lei, 2017; Bastings et al., 2019; Yu et al., 2019; Paranjape et al., 2020; Guerreiro and Martins, 2021; Chan A. et al., 2022
		✓	DeYoung et al., 2020; Hase et al., 2020; Arous et al., 2021; Bhat et al., 2021; Jang and Lukasiewicz, 2021; Mathew et al., 2021
	Pipelined	✗	Zhang et al., 2016; Jiang et al., 2018; Bashier et al., 2020; Jain et al., 2020; Kumar and Talukdar, 2020; Chrysostomou and Aletras, 2022
Attention	Soft-Scores	✗	Vashishth et al., 2019; Wiegreffe and Pinter, 2019
		✓	Bao et al., 2018; Strout et al., 2019; Kanchinadam et al., 2020; Zhang et al., 2021a

#### 2.1.1 Extractive methods

Most research on extractive methods has been carried out using an *encoder-decoder* framework (Lei et al., 2016; DeYoung et al., 2020; Arous et al., 2021). The encoder *enc*(*x*) works as a tagging model, where each word in the input sequence receives a binary tag indicating whether it is included in the rationales *r* (Zaidan et al., 2007). The decoder *dec*(*x, r*) then accepts only the input highlighted as rationales and maps them to one or more target categories (Bao et al., 2018).

The selection of words is performed by an *encoder*, which is a parameterized mapping *enc*(*x*) that extracts rationales from input sequences as *r* = {*x*_*i*_|*z*_*i*_ = 1, *x*_*i*_∈*x*}, where *z*_*i*_∈{0, 1} is a binary tag that indicates whether the word *x*_*i*_ is selected or not. In an extractive setting, the rationale *r* must include only a few words or sentences, and *dec*(*enc*(*x, r*)) should result in nearly the same target vector as the original input when passed through the decoder *dec*(*x*) (Otter et al., 2020; Wang and Dou, 2022).

##### 2.1.1.1 Multi-task models

Lei et al. (2016) pioneered the idea of extracting rationales using the encoder-decoder architecture. They proposed utilizing two models and training them jointly to minimize a cost function composed of a classification loss and sparsity-inducing regularization, responsible for keeping the rationales short and coherent. They identified rationales within the input text by assigning a binary Bernoulli variable to each word. Unfortunately, minimizing the expected cost was challenging since it involved summing over all possible choices of rationales in the input sequence. Consequently, they suggested training these models jointly via REINFORCE-based optimization (Williams, 1992). REINFORCE involves sampling rationales from the encoder and training the model to generate explanations using reinforcement learning. As a result, the model is rewarded for producing rationales that align with desiderata defined in its cost function (Zhang et al., 2021b).

The key components of the solution proposed by Lei et al. (2016) are binary latent variables and sparsity-inducing regularization. As a result, their solution is marked by non-differentiability. Bastings et al. (2019) proposed to replace the Bernoulli variables with rectified continuous random variables, amenable for reparameterization and for which gradient estimation is possible without REINFORCE. Along the same lines, Madani and Minervini (2023) used Adaptive Implicit Maximum Likelihood (Minervini et al., 2023), a recently proposed low-variance and low-bias gradient estimation method for discrete distribution to back-propagate through the rationale extraction process. Paranjape et al. (2020) emphasized the challenges around the sparsity-accuracy trade-off in norm-minimization methods such as the ones proposed by Lei et al. (2016) and Bastings et al. (2019). In contrast, they showed that it is possible to better manage this trade-off by optimizing a bound on the Information Bottleneck objective (Mukherjee, 2019) using the divergence between the encoder and a prior distribution with controllable sparsity levels.

Over the last 15 years, research on *learning with rationales* has established that incorporating human explanations during model training can improve performance and robustness against spurious correlations (Zaidan et al., 2007; Strout et al., 2019). Nonetheless, studies on explainability started addressing how human rationales can also help to enhance the quality of explanations for different NLP tasks (Strout et al., 2019; Arous et al., 2021) only in the past 4 years.

To determine the impact of a supervised approach for extractive rationalization, DeYoung et al. (2020) adapted the implementation of Lei et al. (2016), incorporating human rationales during training by modifying the model's cost function. Similarly, Bhat et al. (2021) developed a multi-task teacher-student framework based on self-training language models with limited task-specific labels and rationales. It is important to note that in the variants of the encoder-decoder architecture using human rationales, the final cost function is usually a composite of the classification loss, regularizers on rationale desiderata, and the loss over rationale predictions (DeYoung et al., 2020; Gurrapu et al., 2023).

One of the main drawbacks of multi-task learning architectures for extractive rationales is that it is challenging to train the encoder and decoder jointly under instance-level supervision (Zhang et al., 2016; Jiang et al., 2018). As described before, these methods sample rationales using regularization to encourage sparsity and contiguity and make it necessary to estimate gradients using either the REINFORCE method (Lei et al., 2016) or reparameterized gradients (Bastings et al., 2019). Both techniques complicate training and require careful hyperparameter tuning, leading to unstable solutions (Jain et al., 2020; Kumar and Talukdar, 2020).

Furthermore, recent evidence suggests that multi-task rationalization models may also incur what is called the degeneration problem, where they produce nonsensical rationales due to the encoder overfitting to the noise generated by the decoder (Madsen et al., 2022; Wang and Dou, 2022; Liu et al., 2023). To tackle this challenge, Liu et al. (2022) introduced a Folded Rationalization approach that folds the two stages of extractive rationalization models into one using a unified text representation mechanism for the encoder and decoder. Using a different approach, Jiang et al. (2023) proposed the YOFO (You Only Forward Once), a simplified single-phase framework with a pre-trained language model to perform prediction and rationalization. It is essential to highlight that rationales extracted using the YOFO framework aim only to support predictions and are not used directly to make model predictions.

##### 2.1.1.2 Pipelined models

Pipelined models are a simplified version of the encoder-decoder architecture in which, first, the encoder is configured to extract the rationales. Then, the decoder is trained separately to perform prediction using only rationales (Zhang et al., 2016; Jain et al., 2020). It is important to note that no parameters are shared between the two models and that rationales extracted based on this approach have been learned in an unsupervised manner since the encoder does not have access to human rationales during training.

To avoid the complexity of training a multi-task learning architecture, Jain et al. (2020) introduced FRESH (Faithful Rationale Extraction from Saliency tHresholding). Their scheme proposed using arbitrary feature importance scores to identify the rationales within the input sequence. An independent classifier is then trained exclusively on snippets the encoder provides to predict target labels. Similarly, Chrysostomou and Aletras (2022) proposed a method that also uses gradient-based scores as the encoder. However, their method incorporated additional constraints regarding length and contiguity for selecting rationales. Their work shows that adding these additional constraints can enhance the coherence and relevance of the extracted rationales, ensuring they are concise and contextually connected, thus improving the understanding and usability of the model in real-world applications.

Going beyond feature importance scores, Jiang et al. (2018) suggested using a reinforcement learning method to extract rationales using a reward function based on latent variables to define the extraction of phrases and classification labels. Their work indicates that reinforcement can optimize the rationale selection process, potentially leading to more accurate explanations by adjusting strategies based on feedback to maximize the reward function. Along the same lines, Guerreiro and Martins (2021) developed SPECTRA (SparsE StruCtured Text Rationalization), a framework based on LP-SparseMAP (Niculae and Martins, 2020). Their method provided a flexible, deterministic and modular rationale extraction process based on a constrained structured prediction algorithm. It is important to note that incorporating a deterministic component can eventually boost the consistency and predictability of the extracted rationales, improving the reliability and reproducibility of explanations across different datasets and applications.

Simplifying the encoder-decoder architecture in extractive rationalization models might enhance its use in explainable NLP systems (Jain et al., 2020; Wang and Dou, 2022). This simplification can lead to more computationally efficient models, broadening their applicability and accessibility in various real-world scenarios.

Recently, there has been increasing interest in leveraging Large Language Models (LLMs) for extractive rationalization, owing to their ability to efficiently process and distill critical information from large text corpora (Wang and Dou, 2022; Gurrapu et al., 2023). The evidence reviewed here suggests that rationalization models might improve performance by prompting language models in a few-shot manner, with rationale-augmented examples. Using this approach, Chen et al. (2023) introduced ZARA, an approach for data augmentation and extractive rationalization using transformer-based models (Vaswani et al., 2017) such as RoBERTa (Liu et al., 2019b), DeBERTa (He et al., 2020), and BART (Lewis et al., 2020). Along the same lines, Zhou et al. (2023) presented a two-stage few-shot learning method that first generates rationales using GPT-3 (Brown et al., 2020), and then fine-tunes a smaller rationalization model, RoBERTa, with generated explanations. It is important to consider a few challenges of using LLMs for rationalization models, including high computational demands and the potential for ingrained biases that can skew language explanations (Zhao et al., 2023).

Even though extractive rationalization may be a crucial component of NLP systems as it enhances trust by providing human-understandable explanations, far too little attention has been paid to its use in real-world applications (Wang and Dou, 2022; Kandul et al., 2023). ExClaim is a good illustration of using extractive rationalization in a high-stake domain. Gurrapu et al. (2022) introduced ExClaim to provide an explainable claim verification tool for use in the legal sector based on extractive rationales that justify verdicts through natural language explanations. Similarly, Mahoney et al. (2022) presented an explainable architecture based on extractive rationales that explain the results of a machine learning model for classifying legal documents. Finally, Tornqvist et al. (2023) proposed a pipelined approach for extractive rationalization to provide explanations for an automatic grading system based on a transformer-based classifier and *post-hoc* explanability methods such as SHAP (Lundberg and Lee, 2017) and Integrated Gradients (Sundararajan et al., 2017).

#### 2.1.2 Attention-based methods

Attention models have not only resulted in impressive performance for text classification (Vaswani et al., 2017), but are also suitable as a potential explainability technique (Vashishth et al., 2019; Wiegreffe and Pinter, 2019). In particular, the attention mechanism has been previously used to identify influential tokens for the prediction task by providing a soft score over the input units (Bahdanau et al., 2015).

Researchers have drawn inspiration from the model architecture from Jain and Wallace (2019) for text classification. For a given input sequence *x*, each token is represented by its *D*-dimensional embedding to obtain $x_{e}\in {\mathbb{R}}^{D\times d}$. Next, a bidirectional recurrent neural network (Bi-RNN) encoder is used to obtain an *m*-dimensional contextualized representation of tokens: $h=Enc\left(x_{e}\right)\in {\mathbb{R}}^{D\times m}$. Finally, the additive formulation of attention proposed by Bahdanau et al. (2015) (*W* ∈ ℝ^*D* × *D*^, *b, c* ∈ ℝ^*D*^ are parameters of the model) is used for computing weights α_*i*_ for all tokens defined as in Equation 1:


(1) $$u_{i}=\tanh(Wh_{i}+b)\;;\;\alpha_{i}=\frac{\exp(u_{i}^{T}c)}{\sum_{j}\left(\exp(u_{j}^{T}c\right)}$$

The weighted instance representation $h_{\alpha }=\overset{T}{\underset{i=1}{\sum }}\alpha_{i}h_{i}$ is fed to a dense layer and followed by a softmax function to obtain prediction $ỹ=\sigma \left(Dec\left(h_{\alpha }\right)\right)\in {\mathbb{R}}^{\left|c\right|}$ where |*c*| denotes the label set size. Finally, a heuristic strategy must be applied to map attention scores to discrete rationales. Examples include selecting spans within a document based on their total score (sum of their tokens' importance scores) or picking the *top-k* tokens with the highest attention scores (Jain et al., 2020).

##### 2.1.2.1 Soft-scores models

Some studies have proposed using variants of attention (Bahdanau et al., 2015) to extract rationales in an unsupervised manner. For explainable text classification, Wiegreffe and Pinter (2019) investigated a model that passes tokens through a BERT model (Devlin et al., 2019) to induce contextualized token representations that are then passed to a bidirectional LSTM (Hochreiter and Schmidhuber, 1997). For soft-score features, they focused attention on the contextualized representation. Similarly, Vashishth et al. (2019) analyzed the attention mechanism on a more diverse set of NLP tasks and assessed how attention enables interpretability through manual evaluation.

Bao et al. (2018) extended the unsupervised approach described above by learning a mapping from human rationales to continuous attention. Like the supervised approach for extractive methods, they developed a model to map human rationales onto attention scores to provide richer supervision for low-resource models. Similarly, Strout et al. (2019) showed that supervising attention with human-annotated rationales can improve both the performance and explainability of results of a classifier based on Convolutional Neural Networks (CNNs; Lai et al., 2015). In the same vein, Kanchinadam et al. (2020) suggested adding a lightweight attention mechanism to a feed-forward neural network classifier and training them using human-annotated rationales as additional feedback.

Even though these are promising methods for extracting rationales, they require access to a significant number of rationale-annotated instances, which might be impractical for domain-specific applications where expert annotators are rare and constrained for time (Vashishth et al., 2019; Kandul et al., 2023). Consequently, Zhang et al. (2021a) proposed HELAS (Human-like Explanation with Limited Attention Supervision). This approach requires a small proportion of documents to train a model that simultaneously solves the text classification task while predicting human-like attention weights. Similarly, Arous et al. (2021) introduced MARTA, a Bayesian framework based on variational inference that jointly learns an attention-based model while injecting human rationales during training. It is important to note that both approaches achieve state-of-the-art results while having access to human rationales for less than 10% of the input documents.

While attention mechanisms have been used for extractive rationalization, their effectiveness as a stand alone explainability method is debated (Burkart and Huber, 2021; Niu et al., 2021). Data from several studies suggest that attention weights might misidentify relevant tokens in their explanations, or they are often uncorrelated with the importance score measured by other explainability methods (Jain and Wallace, 2019; Bastings and Filippova, 2020). This uncertainty has significantly undermined the use of attention-based methods, as they can provide a false sense of understanding of the model's decision-making process, potentially leading to a misguided trust in the NLP system's capabilities and an underestimation of its limitations (Kandul et al., 2023; Lyu et al., 2024).

### 2.2 Abstractive rationale generation

In abstractive rationalization, the aim is to generate natural language explanations to articulate the model's reasoning process describing why an input sequence was mapped to a particular target vector. Abstractive rationales may involve synthesizing or paraphrasing information rather than directly extracting snippets from the input text (Liu et al., 2019a; Narang et al., 2020).

Although extractive rationales are very useful to understand the inner workings of a text classifier, there is a limitation when employing them in tasks that should link commonsense knowledge information to decisions, such as natural language inference (NLI), question-answering, and text classification (Camburu et al., 2018; Rajani et al., 2019). In such cases, rather than extracting relevant words from the input sequence, it is more desirable to provide a more synthesized and potentially insightful overview of the model's decision-making, often resembling human-like reasoning (Liu et al., 2019a; Narang et al., 2020).

There are two main approaches currently being adopted in research into abstractive rationalization: (i) *text-to-text* methods, which rely on sequence-to-sequence translation models such as the Text-to-Text Transfer Transformer (T5) framework proposed by Raffel et al. (2020) including both the label and the explanation at the same time, and (ii) *generative* methods, which first generate a free-form explanation and then makes a prediction based on the produced abstractive rationale (Zhou et al., 2020). Table 2 presents an overview of the methods used to produce abstractive rationales and their representative references.

**Table 2:** Overview of common approaches for abstractive rationale generation.

**Approach**	**Method**	**Supervised**	**Representative paper(s)**
Text-to-text	Multi-task	✓	Narang et al., 2020; Jang and Lukasiewicz, 2021
Generative	Pipelined	✓	Kumar and Talukdar, 2020; Zhao and Vydiswaran, 2021
	Multi-task	✓	Liu et al., 2019a; Atanasova et al., 2020; Camburu et al., 2020; Zhou et al., 2020; Li et al., 2021

It is important to note that a relatively small body of literature is concerned with abstractive rationalization for explainable text classification. Abstractive rationales are used less frequently than extractive rationales primarily due to the higher complexity and technical challenges in generating coherent, accurate, and relevant synthesized explanations (Madsen et al., 2022; Ji et al., 2023). Consequently, most of the studies on abstractive rationalization have been based on supervised methods, where human explanations are provided during the model's training (Liu et al., 2019a; Zhou et al., 2020).

#### 2.2.1 Text-to-text methods

A text-to-text model follows the sequence-to-sequence (seq2seq) framework (Sutskever et al., 2014), where it is fed a sequence of discrete tokens as input and produces a new sequence of tokens as output. Using this approach, researchers have leveraged the T5 framework to train a joint model designed to generate explanations and labels simultaneously (Raffel et al., 2020). Consequently, a model is fit to maximize the following conditional likelihood of the target label *y* and explanations *e* given the input text *x* as defined in Equation 2:


(2) $$\begin{array}{l} L=\overset{n}{\underset{i=1}{\prod }}p\left(y_{i},e_{i}|x_{i}\right) \end{array}$$

##### 2.2.1.1 Multi-task models

Text-to-text methods for generating abstractive rationales leverage the text-to-text framework proposed by Raffel et al. (2020) to train language models to output natural text explanations alongside their predictions. A study by Narang et al. (2020) showed that their WT5 model (T5 models using “base” and “11B” configurations; Raffel et al., 2020) achieved state-of-the-art results with respect to the quality of explanations and classification performance, when having access to a relatively large set of labeled examples. Finally, they also claimed that their WT5 model could help transfer a model's explanation capabilities across different data sets.

Similarly, Jang and Lukasiewicz (2021) conducted experiments evaluating abstractive rationales generated by a T5-base model for text classification and NLI. Nevertheless, their work emphasized the need to reduce the volume of rationale-annotated data and the computational requirements required to train these models to produce comprehensive and contextually appropriate rationales.

Text-to-text models have shown promising results for improving the understanding of classification models and increasing the prediction performance using explanations as additional features (Gilpin et al., 2018; Danilevsky et al., 2020). However, their training requires a large number of human-annotated rationales. This property precludes the development of free-text explainable models for high-stake domains where rationale-annotated data sets are scarcely available (Jang and Lukasiewicz, 2021).

#### 2.2.2 Generative methods

Researchers investigating generative methods have utilized a *generator-decoder* framework (Camburu et al., 2018; Rajani et al., 2019), which is similar to the *encoder-decoder* used for extractive rationalization. The generator *gen*(*x*) works as a seq2seq model where each input sequence is mapped onto a free-form explanation (Zhou et al., 2020). The decoder *dec*(*x*) then takes the abstractive rationale to predict the target vector (Jang and Lukasiewicz, 2021).

By using the multiplication law of conditional probability, we can decompose Equation (3) and formulate the training of generative methods as Zhou et al. (2020):


(3) $$\begin{array}{l} L=\overset{n}{\underset{i=1}{\prod }}\underset{\text{Generator}}{\underset{︸}{p\left(e_{i}|x_{i}\right)}}\underset{\text{Decoder}}{\underset{︸}{p\left(y_{i}|x_{i},e_{i}\right)}} \end{array}$$

An explanation generator model *gen*(*x*) that parameterizes *p*(*e*_*i*_|*x*_*i*_) takes an input sequence *x* and generates a corresponding natural language explanation *e*. As mentioned, the abstractive rationale might not be found in the input sequence *x* (Zhou et al., 2020). The decoder *dec*(*x, e*) is an augmented prediction model, which parameterizes *p*(*y*_*i*_|*x*_*i*_, *e*_*i*_) and takes an input sequence *x* and an explanation *e* to assign a target vector *y* (Rajani et al., 2019; Atanasova et al., 2020).

A significant advantage of generative methods for abstractive rationalization is that they require significantly fewer human-annotated examples for training an explainable text classification model than text-to-text methods. Due to their flexibility in creating new content, generative methods allow for a broader range of expressive and contextually relevant rationales that can closely mimic human-like explanations (Liu et al., 2019a; Zhou et al., 2020).

##### 2.2.2.1 Pipelined models

As with extractive methods, pipelined models for abstractive rationalization simplify the generator-decoder architecture. Both modules are trained independently, with no parameters shared between the two models. Kumar and Talukdar (2020) proposed a framework where a pre-trained language model based on the GPT-2 architecture (Radford et al., 2019) is trained using a causal language modeling loss (CLM). An independent RoBERTa-based (Liu et al., 2019b) classifier is then fit on the abstractive rationales to predict target labels. Similarly, Zhao and Vydiswaran (2021) introduced LiREX, a framework also based on a GPT-2-based generator and a decoder leveraging RoBERTa. However, this framework included an additional component at the start of the pipeline that first extracts a label-aware token-level extractive rationale and employs it to generate abstractive explanations. Due to the possibility of generating label-aware explanations, LiREX is especially suitable for multi-label classification problems.

##### 2.2.2.2 Multi-task models

Drawing inspiration from the work of Camburu et al. (2018) on abstractive rationalization for explainable NLI, Zhou et al. (2020) developed the ELV (Explanations as Latent Variables) framework. They used a variational expectation-maximization algorithm (Palmer et al., 2005) for optimization where an explanation generation module and an explanation-augmented BERT module are trained jointly. They considered natural language explanations as latent variables that model the underlying reasoning process of neural classifiers. Since training a seq2seq model to generate explanations from scratch is challenging, they used UniLM (Dong et al., 2019), a pre-trained language generation model, as the generation model in their framework. Similarly, Li et al. (2021) proposed a joint neural predictive approach to predict and generate abstractive rationales and applied it to English and Chinese medical documents. As generators, they used the large version of T5 (T5 large; Raffel et al., 2020) and its multilingual version, mT5 (Xue et al., 2021). For classification, they applied ALBERT (Lan et al., 2019) and RoBERTa (Liu et al., 2019b) on the English and Chinese data sets, respectively. Even though they found that the multi-task learning approach boosted model explainability, the improvement in their experiments was not statistically significant.

A few studies have shown that generative methods sometimes fail to build reliable connections between abstractive rationales and predicted outcomes (Carton et al., 2020; Wiegreffe et al., 2021). Therefore, there is no guarantee that the generated explanations reflect the decision-making process of the prediction model (Tan, 2022). To generate faithful explanations, Liu et al. (2019a) suggested using an explanation factor to help build stronger connections between explanations and predictions. Their Explanation Factor (EF) considers the distance between the generated and the gold standard rationales and the relevance between the abstractive rationales and the original input sequence. Finally, they included EF in the objective function and jointly trained the generator and decoder to achieve state-of-the-art results for predicting and explaining product reviews.

New findings amongst abstractive rationalization provide further evidence that models are prone to hallucination (Kunz et al., 2022; Ji et al., 2023). In explainable text classification, hallucination refers to cases where a model produces factually incorrect or irrelevant rationales, thus impacting the reliability and trustworthiness of these explanations (Zhao et al., 2023). Even though most evaluation metrics punish hallucination and try to mitigate it during training, the irrelevant rationales included might add helpful information for the classification step and, therefore, be used regardless. This phenomenon can mislead users about the model's decision-making process, undermining the credibility of NLP systems and posing challenges for its practical application in scenarios requiring high accuracy and dependability (Wang and Dou, 2022; Ji et al., 2023).

Zero-shot approaches are increasingly relevant in NLP as they allow models to process language tasks they have not been explicitly trained on, enhancing their adaptability as part of real-world solutions where training data is not necessarily available (Meng et al., 2022). Even though there is a relatively small body of literature that is concerned with zero-shot rationalization approaches for explainable text classification, studies such as that conducted by Kung et al. (2020) and Lakhotia et al. (2021) have shown that zero-shot rationalization models achieve comparable performance without any supervised signal. Nevertheless, a significant challenge is the model's ability to produce relevant rationales for unseen classes, as it must extrapolate from learned concepts without direct prior knowledge (Lyu et al., 2021). This capability requires understanding abstract and transferable features across different contexts, difficulting the training and deployment of these rationalization models (Wei et al., 2021; Meng et al., 2022). It is important to note that, if successful, they can enhance the scalability of NLP systems by making them capable of analyzing data from various domains without needing extensive retraining (Kung et al., 2020; Yuan et al., 2024).

## 3 Rationale-annotated datasets

During the last 15 years, there has been an increase in the volume of rationale-annotated data available, boosting progress on designing more explainable classifiers and facilitating the evaluation and benchmarking of rationalization approaches (DeYoung et al., 2020; Wang and Dou, 2022).

Table 3 describes each rationale-annotated dataset for text classification in terms of their domain, the annotation procedure used to collect the human explanations (indicated as “author” or “crowd” for crowd-annotated), their number of instances (input-label pairs), their publication year and the original paper where they were presented. Moreover, it includes links to each dataset (when available), providing direct access for further exploration and detailed analysis.

**Table 3:** Comparison of rationale-annotated datasets for text classification.

**Dataset name**	**Domain**	**Collection**	**Instances**	**Year**	**References**
MovieReviews (v.1.0)	Product reviews	Author	2,000	2007	Zaidan et al., 2007
AmazonReviews	Product reviews	Crowd	6,000	2007	Blitzer et al., 2007
HotelReviews	Product reviews	Crowd	109,000	2010	Wang et al., 2010
Nova	Social media	Crowd	12,000	2011	Guyon et al., 2011
IMDB	Product reviews	Crowd	25,000	2011	Maas et al., 2011
BeerAdvocate	Product reviews	Crowd	4,000	2012	McAuley et al., 2012
SST	social media	crowd	11,855	2013	Socher et al., 2013
WikiAttack	Social media	Author	1,089	2018	Carton et al., 2018
FEVER	Social media	Crowd	136,000	2018	Thorne et al., 2018
MovieReviews (v.2.0)	Product reviews	Crowd	200	2019	DeYoung et al., 2020
Snopes Corpus	Social media	Crowd	6,422	2019	Hanselowski et al., 2019
HateXplain	Social media	Crowd	20,148	2020	Mathew et al., 2021
Yelp-HAT	Product reviews	Crowd	15,000	2020	Sen et al., 2020
RaFoLa	Modern slavery	Author	989	2021	Mendez et al., 2022
Hummingbird	Social media	Crowd	500	2021	Hayati et al., 2021
SBIC	Social media	Author	360	2022	Marasović et al., 2022
DynaSent	Product reviews	Author	2,880	2023	Jakobsen et al., 2023

Incorporating human rationales during training of supervised learning models can be traced back to the work of Zaidan et al. (2007), where a human teacher highlighted text spans in a document to improve model performance. Their MovieReviews(v.1.0) corpus is the first rationale-annotated dataset for text classification, including 1,800 positive/negative sentiment labels on movie reviews.

Table 3 shows that the dominant collection paradigm is via crowd sourcing platforms. A critical bottleneck of rationale generation is the insufficient domain-specific rationale-annotated data (Lertvittayakumjorn and Toni, 2019). Gathering enough *(input, label, and human rationales*) triples from potential end-users is essential as it provides rationalization models with a reference for what constitutes a meaningful and understandable explanation from a human perspective (Strout et al., 2019; Carton et al., 2020; DeYoung et al., 2020). Rationale-annotated data is critical in real-world applications, where the alignment of machine-generated rationales with human reasoning greatly enhances the model's transparency, trustworthiness, and acceptance by users in practical scenarios (Wang and Dou, 2022; Gurrapu et al., 2023).

Creating benchmark data sets with human annotations is essential for training and comparing rationalization models, as they provide a standardized resource to evaluate the effectiveness, accuracy, and human-likeness of model-generated explanations (Jacovi and Goldberg, 2021; Wang and Dou, 2022). Such benchmarks facilitate consistent, objective comparison across different models, fostering advancements in the field by highlighting areas of strength and opportunities for improvement in aligning machine-generated explanations with human reasoning and understanding (Kandul et al., 2023; Lyu et al., 2024). The task of extractive rationalization was surveyed by DeYoung et al. (2020), who proposed the ERASER (Evaluating Rationales And Simple English Reasoning) benchmark spanning a range of NLP tasks. These data sets, including examples for text classification such as MovieReviews(v.2.0) and FEVER, have been repurposed from pre-existing corpora and augmented with labeled rationales (Zaidan et al., 2007; Thorne et al., 2018). More recently, Marasović et al. (2022) introduced the FEB benchmark containing four English data sets for few-shot rationalization models, including the SBIC corpus for offensiveness classification.

Questions have been raised about using human-annotated rationales for training and evaluating rationalization models since they are shown to be quite subjective (Lertvittayakumjorn and Toni, 2019; Carton et al., 2020). Most published studies failed to specify information about the annotators, such as gender, age, or ethnicity. Jakobsen et al. (2023) makes an essential contribution by being the first dataset to include annotators' demographics and human rationales for sentiment analysis. Diversity in collecting human rationales is crucial to the development of universally understandable and reliable models, enhancing their applicability and acceptance across a broad spectrum of stakeholders and scenarios (Tan, 2022; Yao et al., 2023).

Finally, different methods have been proposed to collect human rationales for explainable text classification. On the one hand, in some studies (e.g., Zaidan et al., 2007), annotators were asked to identify *the most important* phrases and sentences supporting a label. On the other hand, in the work of Sen et al. (2020), for example, *all* sentences relevant to decision-making were identified. Even though these approaches seem similar, they might lead to substantially different outcomes (Hartmann and Sonntag, 2022; Tan, 2022). Documentation and transparency in the annotation of human rationales are essential as they provide clear insight into the reasoning process and criteria used by human annotators, ensuring replicability and trustworthiness in the model evaluation process (Carton et al., 2020). This detailed documentation is crucial for understanding potential biases and the context under which these rationales were provided, thereby enhancing the credibility and generalizability of the rationalization models.

## 4 Evaluation metrics

The criteria for evaluating the quality of rationales in explainable text classification are not universally established. Generally, evaluation approaches fall into two categories: (i) *proxy-based*, where rationales are assessed based on automatic metrics that attempt to measure different desirable properties (Carton et al., 2020; DeYoung et al., 2020), and (ii) *human-grounded*, where humans evaluate rationales in the context of a specific application or a simplified version of it (Doshi-Velez and Kim, 2017; Lertvittayakumjorn and Toni, 2019).

Table 4 summarizes the categories for rationale evaluation, including metrics and their most relevant references.

**Table 4:** Overview of evaluation metrics for rationale's quality.

**Approach**	**Desiderata**	**Representative paper(s)**
Proxy-based	Plausibility	Paranjape et al., 2020; Guerreiro and Martins, 2021; Jang and Lukasiewicz, 2021; Chan A. et al., 2022; Atanasova et al., 2024
	Faithfulness	Carton et al., 2020; DeYoung et al., 2020; Zhang et al., 2021a; Chan A. et al., 2022
	Simulatability	Hase et al., 2020
	Consistency	Atanasova et al., 2024
	Robustness	Chen H. et al., 2022; Ross et al., 2022
Human-grounded	Understandability	Ehsan et al., 2019; Lertvittayakumjorn and Toni, 2019; Hase and Bansal, 2020; Jain et al., 2020
	Relatability	Ehsan et al., 2019; Lertvittayakumjorn and Toni, 2019; Hase and Bansal, 2020

### 4.1 Proxy-based

*Plausibility* in rationalization for text classification refers to the extent to which explanations provided by a model align with human intuition and understanding (DeYoung et al., 2020; Wiegreffe et al., 2021). Plausible explanations enhance the trust and credibility of classifiers, as they are more likely to be understood and accepted by end-users, particularly those without technical expertise (Doshi-Velez and Kim, 2017; Hase and Bansal, 2022; Atanasova et al., 2024). DeYoung et al. (2020) proposed evaluating plausibility using Intersection-over-Union at the token level to derive token-level precision, recall, and F1 scores. Several studies have followed a similar evaluation approach for extractive rationalization models (Paranjape et al., 2020; Guerreiro and Martins, 2021; Chan A. et al., 2022), while others have explored using phrase-matching metrics such as SacreBLEU and METEOR (Jang and Lukasiewicz, 2021) for evaluating abstractive rationales. In the case of attention-based methods that perform soft selection, DeYoung et al. (2020) suggested measuring plausibility using the Area Under the Precision-Recall Curve (AUPRC) constructed by sweeping a threshold over token scores (DeYoung et al., 2020; Chan A. et al., 2022).

While plausibility is important for rationalization models, much of the literature acknowledges that generating plausible rationales is not enough (Doshi-Velez and Kim, 2017; Arrieta et al., 2020; Danilevsky et al., 2020). Previous research has established that it is crucial to ensure that the rationales also reflect the actual reasoning processes of the model rather than being superficial or misleading (Belle and Papantonis, 2021; Jacovi and Goldberg, 2021). *Faithfulness* refers to the degree to which the generated rationales accurately represent the internal decision-making process of the model. DeYoung et al. (2020) proposed two automatic metrics for assessing faithfulness by measuring the impact of perturbing or erasing snippets within language explanations. First, *comprehensiveness* captures the extent to which all relevant features for making a prediction were selected as rationales. Second, *sufficiency* assesses whether the snippets within rationales are adequate for a model to make a prediction. Using this approach, researchers have established that a faithful rationale should have high comprehensiveness and sufficiency (Zhang et al., 2021a; Chan A. et al., 2022).

Supporting this view, Carton et al. (2020) introduced the term *fidelity* to refer jointly to sufficiency and comprehensiveness. According to their findings, a rationale can contain many tokens irrelevant to the prediction while still having high comprehensiveness and low sufficiency. Consequently, they introduced the idea of *fidelity curves* to assess rationale irrelevancy by looking at how sufficiency and comprehensiveness degrade as tokens are randomly occluded from a language explanation. There is a consensus among researchers and practitioners that this level of authenticity in explanations is crucial for users to scrutinize NLP decisions, particularly in high-stake domains where understanding the model's reasoning is paramount (Miller, 2019; Tjoa and Guan, 2020; Bibal et al., 2021).

*Robustness* refers to the model's ability to consistently provide reliable rationales across various inputs and conditions (Gunning et al., 2019; Arrieta et al., 2020; Lyu et al., 2024). Robustness is crucial for explainable text classification as it ensures dependability and generalizability of the explanations, particularly in real-world applications where data variability and unpredictability are common (Belle and Papantonis, 2021; Hartmann and Sonntag, 2022). Most researchers investigating robustness in rationalization models have utilized adversarial examples to evaluate the model's rationales to remain trustworthy and reliable in potentially deceptive environments (Zhang et al., 2020; Liang et al., 2022). Using this approach, Chen H. et al. (2022) assessed the model's robustness by measuring performance on challenge datasets where human-annotated edits to inputs that can change classification labels, are available. Similarly, Ross et al. (2022) proposed assessing robustness by testing whether rationalization models are invariant to adding additional sentences and remain consistent with their predictions. Data from both studies suggest that rationalization models can improve robustness. However, leveraging human rationales as extra supervision does not always translate to more robust models.

It is important to note that most rationale evaluation research has focused on extractive rationalization models (Carton et al., 2020; Hase and Bansal, 2020). Assessing abstractive rationales for explainable text classification presents several unique challenges. First, the subjective nature of abstractive rationales makes standardization of evaluation metrics, such as plausibility difficult, as these rationales do not necessarily align with references of the original input text (Camburu et al., 2020; Zhao and Vydiswaran, 2021). Second, ensuring faithfulness and robustness of abstractive rationales is complex, as they involve generating new text that may not directly correspond to specific input features, making it challenging to determine whether the rationale reflects the model's decision-making reliably (Dong et al., 2019; Zhou et al., 2020). These challenges highlight the need for innovative and adaptable evaluation frameworks that can effectively capture the multifaceted nature of abstractive rationales in explainable NLP systems.

### 4.2 Human-grounded

Even though the vast majority of research on rationale evaluation has been proxy-based, some studies have begun to examine human-grounded evaluations for explainable text classification (Mohseni et al., 2018; Ehsan et al., 2019). Nevertheless, to our knowledge, there is no published research on human-grounded methods using domain experts in the same target application. Instead, we have found some studies conducting simpler human-subject experiments that maintain the essence of the target application.

According to Ehsan et al. (2019), rationale *understandability* refers to the degree to which a rationale helped an observer understand why a model behaved as it did. They asked participants to rate the understandability of a set of rationales using a 5-point Likert scale. Instead, Lertvittayakumjorn and Toni (2019) used binary forced-choice experiments. As part of their research, humans were presented with pairs of explanations to choose the one they found more understandable.

Finally, researchers have also been interested in measuring *simulatability* using human-subject simulation experiments. In a qualitative study by Lertvittayakumjorn and Toni (2019), humans were presented with input-explanation pairs and asked to simulate the model's outcomes correctly. Similarly, Ehsan et al. (2019) assessed simulatability using counterfactual simulation experiments. In this case, observers were presented with input-output-explanation triples and asked to identify what words needed to be modified to change the model's prediction to the desired outcome.

In an investigation into human-grounded metrics for evaluating rationales in text classification, Lertvittayakumjorn and Toni (2019) concluded that experiments and systems utilized to collect feedback on machine-generated rationales lack interactivity. In almost every study, users cannot contest a rationale or ask the system to explain the prediction differently. This view is supported by Ehsan et al. (2019), who concluded that current human-grounded experiments could only partially assess the potential implications of language explanations in real-world scenarios.

Even though human-grounded evaluation is key in assessing the real-world applicability and effectiveness of rationalization models, it presents several challenges that stem from the inherent subjectivity and variability of human judgment (Doshi-Velez and Kim, 2017; Carton et al., 2020). First, the diversity of interpretations among different evaluators can lead to an inconsistent assessment of the quality and relevance of the generated rationales (Lertvittayakumjorn and Toni, 2019; Hase and Bansal, 2020). As mentioned before, this diversity is influenced by cultural background, domain expertise, and personal biases, making it difficult to consolidate a standardized evaluation metric (Mohseni et al., 2018; Yao et al., 2023). Second, the cognitive load on human evaluators can be significant, especially when dealing with complex classification tasks or lengthy rationales, potentially affecting the consistency and reliability of their judgment (Tan, 2022). Finally, there is the scalability challenge, as human evaluations are time-consuming and resource-intensive, limiting the feasibility of conducting large-scale assessments (Kandul et al., 2023).

## 5 Challenges and future outlook

In this section, we discuss the current challenges in developing trustworthy rationalization models for explainable text classification and suggest possible approaches to overcome them.

### 5.1 Rationalization approaches

Extractive and abstractive rationalization approaches have distinct advantages and disadvantages when applied to explainable text classification. Table 5 summarizes the trade-offs of the rationalization methods described in Section 2.

**Table 5:** Main advantages and disadvantages of methods for rationale generation.

**Rationale**	**Approach**	**Advantages**	**Disadvantages**
Extractive	Extractive	Works with limited or no rationale-annotated data	Hard to train leading to unstable outcomes
	Attention	Achieves good classification performance	Risks of identifying unreliable rationales
Abstractive	Text-to-text	Produces comprehensive rationales	Require large amounts of rationale-annotated data
	Generative	Works with a limited amount of human rationales	Possible misalignment between rationales and labels

Extractive rationalization, which involves selecting parts of the input text as justification for the model's decision, boasts the advantage of being directly linked to the original data, often making these explanations more straightforward and more accessible to validate for accuracy (Wang and Dou, 2022; Gurrapu et al., 2023). However, this method can be limited in providing context or explaining decisions requiring synthesizing information not explicitly stated in the text (Kandul et al., 2023; Lyu et al., 2024). Abstractive rationalization, which generates new text to explain the model's decision, offers greater flexibility and can provide more holistic and nuanced explanations that synthesize various aspects of the input data. This approach can be more intuitive and human-like, enhancing the comprehensibility for end-users (Li et al., 2021; Zini and Awad, 2022). Yet, it faces challenges such as the risk of hallucination—producing explanations that are not grounded in the input data—and the complexity of ensuring that these generated explanations are both accurate and faithful to the model's decision-making process (Liu et al., 2019a; Hase and Bansal, 2020). Therefore, while extractive methods offer reliability and direct traceability, abstractive methods provide richness and depth, albeit with increased challenges in maintaining fidelity and accuracy (Wiegreffe et al., 2021; Yao et al., 2023).

The choice between extractive and abstractive rationalization models for explainable text classification largely depends on the specific requirements and constraints of the application (Wang and Dou, 2022; Gurrapu et al., 2023). On the one hand, extractive rationalization models are generally more suitable in scenarios where transparency and direct traceability to the original text are paramount. They are ideal when the rationale for a decision needs to be anchored to specific parts of the input text, such as in legal or compliance-related tasks where every decision must be directly linked to particular evidence or clauses (Bibal et al., 2021; Lyu et al., 2024). On the other hand, abstractive rationalization models are better suited for scenarios where a more synthesized understanding or a broader context is necessary (Miller, 2019; Kandul et al., 2023). They excel in situations where the rationale might involve drawing inferences or conclusions not explicitly stated in the text. Abstractive models are also preferable when the explanation needs to be more accessible to laypersons, as they can provide more natural, human-like explanations (Amershi et al., 2014; Tjoa and Guan, 2020).

Even though the decision to use pipelined or multi-task learning models for rationalization depends on the specific goals and constraints, several studies suggest that multi-task learning models perform better for both extractive and abstractive rationalization (Dong et al., 2019; Zhou et al., 2020; Li et al., 2021; Wang and Dou, 2022). Pipelined models are advantageous when each module, rationalization and classification, require specialized handling or when modularity is needed in the system (Jain et al., 2020; Chrysostomou and Aletras, 2022). This approach allows for greater flexibility in updating each component independently. However, they can suffer from error propagation where the rationalization can affect the classification (Kunz et al., 2022). In contrast, multi-task learning models are generally more efficient and can offer performance benefits, enabling sharing of insights between tasks. Nevertheless, they may require more training data, more complex hyperparameter tuning and careful balancing of the learning objectives (Bastings et al., 2019; Chan A. et al., 2022). Finally, the choice depends on the specific requirements for model performance, the availability of training data, and the need for flexibility in model deployment and maintenance.

Since approaches have been trained and tested on different datasets using a variety of evaluation metrics, we have ranked them based on their reported performance on the MovieReviews (Zaidan et al., 2007), SST (Socher et al., 2013), and FEVER (Thorne et al., 2018) datasets. Table 6 compares the performance of each rationalization approach in terms of its predictive performance and the quality of its produced rationales using sufficiency and comprehensiveness scores. Based on the results reported by the authors, we have categorized the predictive performance into: ✓✓✓—Very good performance, ✓✓—Good performance, and ✓— Performance has potential for improvement. What stands out in this table is the dominance of multi-task methods over pipelined and soft-score approaches in terms of predictive performance and explainability. Our summary shows that supervised multi-task extractive approaches are state-of-the-art for rationalization in terms of predictive performance and rationales' quality, followed by supervised multi-task text-to-text abstractive methods. We refer the reader to bf for details of each rationalization approach's performance.

**Table 6:** Summary of the evaluation of each rationalization approach in terms of its predictive capability and the quality of its generated explanations.

**Rationalization**	**Approach**	**Method**	**Supervision**	**Predictive performance**	**Explanation quality**
Extractive	Extractive	Multi-task	Unsupervised	✓✓	✓✓
			Supervised	✓✓✓	✓✓✓
		Pipelined	Unsupervised	✓	✓
	Attention	Soft-scores	Unsupervised	✓✓	✓
			Supervised	✓✓	✓✓
Abstractive	Text-to-text	Multi-task	Supervised	✓✓✓	✓✓
		Pipelined	Supervised	✓✓	✓
	Generative	Multi-Task	Supervised	✓✓	✓✓

Combining extractive and abstractive rationales for explainable text classification represents an innovative approach that harnesses the strengths of both: the direct, evidence-based clarity of extractive rationales and the comprehensive, context-rich insights of abstractive explanations. A recent study by Majumder et al. (2022) introduced RE*x*C (Extractive Rationales, Natural Language Explanations, and Commonsense), a rationalization framework that explains its prediction using a combination of extractive and abstractive language explanations. RE*x*C selects a subset of the input sequence as an extractive rationale using an encoder based on the HardKuma distribution (Bastings et al., 2019), passes the selected snippets to a BART-based generator (Lewis et al., 2020), and inputs the abstractive rationales to a decoder that outputs the final prediction. It is essential to highlight that all models are trained jointly, and the supervision comes from the target vectors and human-annotated explanations.

Beyond unimodal rationalization models for explainable text classification, multimodal explanations, which integrate textual, visual, and sometimes structured information, can provide more comprehensive insights into AI models' decision-making processes (Park et al., 2018). Using this approach, Marasović et al. (2020) have produced abstractive rationales for visual reasoning tasks, such as visual-textual entailment, by combining pre-trained language models with object recognition classifiers to provide image understanding at the semantic and pragmatic levels. Along the same lines, Zhang et al. (2024) developed a vision language model to identify emotions in visual art and explain their prediction through abstractive rationales. Recent evidence suggests that multimodal explanations can allow for a deeper understanding of how different types of data can be analyzed to produce more accessible and intuitive explanations, broadening the scope and applicability of rationalization in real-world scenarios (Chen and Zhao, 2022; Ananthram et al., 2023; Zhang et al., 2024).

### 5.2 Rationale-annotated data

Generating more rationale-annotated data is crucial for training and evaluating rationalization models, as it provides a rich, diverse foundation for teaching these models how to produce relevant and human-understandable explanations (Doshi-Velez and Kim, 2017; Hase and Bansal, 2020). These data sets enhance the model's ability to generate accurate and more contextually appropriate rationales and facilitate more robust and comprehensive evaluation, improving the model's reliability and effectiveness in real-world applications. Even though there has been vast progress since the publication of ERASER (DeYoung et al., 2020) and FEB (Marasović et al., 2022) benchmarks, there is still a lack of rationale-annotated data for text classification. Considering that highlighting human rationales is not significantly more expensive than traditional labeling (Zaidan et al., 2007), the NLP community could move toward methods for collecting labels by annotating rationales. By doing so, we could boost the results of classification and rationalization models (Arous et al., 2021).

However, it is not enough to have more rationale-annotated data. We also need better human rationales. Standardizing methods for collecting rationale-annotated data is pivotal in the development of rationalization models, as it ensures a uniform approach to gathering and interpreting data, crucial for maintaining the quality and consistency of training and evaluation processes (Wiegreffe et al., 2021; Yao et al., 2023). Documenting and reporting these procedures is equally important, providing transparency about how the data was annotated and allowing applicability in future research (Atanasova et al., 2020; Li et al., 2021). Moreover, reporting and fostering the diversity of the annotators involved is critical. Diversity in demographics, expertise, and cognitive perspectives significantly shape machine-generated rationales (Jakobsen et al., 2023). A comprehensive approach to data annotation is vital to advancing rationalization models that are reliable, effective and ethically sound in their explanations, catering to a broad spectrum of real-world applications and stakeholders.

Further work is needed to establish whether crafting datasets annotated with multimodal explanations can enrich the training and capabilities of rationalization approaches for explainable NLP. Even though preliminary results seem to indicate those visual and textual rationales can indeed provide explanatory strengths (Chen and Zhao, 2022; Ananthram et al., 2023), one of the main challenges is the complexity involved in integrating diverse data types to ensure that annotations reflect the interconnectedness of these modalities (Marasović et al., 2020). Moreover, developing robust annotation guidelines that capture the nuances of multimodal interactions is complex and requires interdisciplinary expertise (Yuan et al., 2024; Zhang et al., 2024).

Since the reasoning process needed to infer a label is subjective and unstructured, we must develop dynamic, flexible and iterative strategies to collect human rationales (Doshi-Velez and Kim, 2017). Considering that we aim to describe the decision-making process in real-world applications accurately, we could move toward noisy data labeling processes attempting to reflect the annotator's internal decision procedure. To illustrate, if annotators change their minds while highlighting rationales, dynamic approaches should be able to capture these changes so that we can learn from them (Ehsan et al., 2019). This dynamic approach might allow for a more authentic and comprehensive representation of human cognitive processes, enriching the training and evaluation of rationalization models with insights that mirror the nature of real-world human thought and decision-making.

The use of human rationales has been key to the development of explainable text classification models. However, further research should focus on whether humans can provide explanations that can later be used to train rationalization models (Miller, 2019; Tan, 2022). We need to acknowledge that human rationales, while a valid proxy mechanism, can only help us to understand the decision-making process of humans partially (Amershi et al., 2014). Consequently, we encourage the NLP community to stop looking at them as another set of uniform labels and embrace their complexity by working collaboratively with researchers in other domains. For instance, to understand whether data sets of human explanations can serve their intended goals in real-world applications, we must connect the broad range of notions around human rationales in NLP with existing psychology and cognitive science literature. A more holistic understanding of human explanations should allow us to decide what kind of explanations are desired for NLP systems and help clarify how to generate and use them appropriately within their limitations.

### 5.3 Comprehensive rationale evaluation

While significant progress has been made in evaluating rationalization models, areas require improvement to ensure safer and more sustainable evaluation (Lertvittayakumjorn and Toni, 2019; Carton et al., 2020). Even though current approaches offer valuable insights, there is a need for evaluation frameworks that can assess the suitability and usefulness of the rationales in diverse and complex real-world scenarios (Chen H. et al., 2022; Hase and Bansal, 2022). Additionally, there is a growing need to focus on the ethical implications of rationale evaluation, particularly in sensitive applications (Atanasova et al., 2023; Joshi et al., 2023). As a community of researchers and practitioners, we must ensure that the models do not inadvertently cause harm or perpetuate misinformation. Addressing these challenges requires a concerted effort from the XAI community to innovate and collaborate, paving the way for more reliable, fair, and transparent rationalization models in NLP.

We have provided a list of diagnostic properties for assessing rationales. It is important to note that these evaluation metrics have mainly been generated from a developer-based perspective, which has biased their results toward faithful explanations (Lertvittayakumjorn and Toni, 2019; DeYoung et al., 2020). Current evaluation approaches are not designed nor implemented considering the perspective of other relevant stakeholders, such as investors, business executives, end-users, and policymakers, among many others. Further work must be done to evaluate rationale quality from a broader perspective, including practical issues that might arise in their implementation for real-world applications (Tan, 2022).

Considering how important language explanations are for building trust with end-users (Belle and Papantonis, 2021), their contribution should also be evaluated in the context of their specific application (Doshi-Velez and Kim, 2017). A lack of domain-specific annotated data is detrimental to developing explainable models for high-stake sectors such as the legal, medical and humanitarian domains (Jacovi and Goldberg, 2021; Mendez et al., 2022). As mentioned before, current evaluation methods lack interactivity (Carton et al., 2020). End users or domain experts cannot contest rationales or ask the models to explain them differently, which makes them impossible to validate and deploy in real-world applications. Even though it is beyond the scope of our survey, work needs to be done to develop clear, concise and user-friendly ways of presenting rationales as part of explainable NLP systems (Hartmann and Sonntag, 2022; Tan, 2022). Effectively communicated rationales boost user trust and confidence in the system and facilitate a deeper comprehension of the model's decision-making process, leading to more informed and effective use of NLP models.

## 6 Conclusions

Developing understandable and trustworthy systems becomes paramount as NLP and text classification applications continue to integrate into critical and sensitive applications. The present survey article aimed to examine rationalization approaches and their evaluation metrics for explainable text classification, providing a comprehensive entry point for new researchers and practitioners in the field.

The contrast between extractive and abstractive rationalization highlights distinct strengths and limitations. On the one hand, extractive rationalization approaches link to original data, ensuring reliability and ease of validation. However, they may lack the context or comprehensive insight needed for decision-making. On the other hand, abstractive rationalization models offer the flexibility to produce more intuitive and human-like explanations, which enhance user usability and trust. Nevertheless, they face challenges such as the potential for generating non-factual explanations and the complexity of maintaining plausibility in the decision-making process. Choosing between extractive and abstractive models depends on application-specific needs: extractive models are preferable where direct traceability is crucial, such as legal applications. In contrast, abstractive models are suited for situations requiring broader contextual interpretations.

Despite its challenging nature, the emerging work on rationalization for explainable text classification is promising. Nevertheless, several questions remain to be answered. Further research is required to better understand human rationales, establish procedures for collecting them, and develop accurate and feasible methods for generating and evaluating rationales in real-world applications. We have identified possible directions for future research, which will hopefully extend the work achieved so far.

## Author contributions

EM: Conceptualization, Investigation, Methodology, Validation, Visualization, Writing – original draft, Writing – review & editing. VS: Conceptualization, Supervision, Writing – review & editing. RB-N: Conceptualization, Supervision, Writing – review & editing.

## Appendix

### Performance of rationalization approaches

Table A1 presents the breakdown results for rationalization approaches according to what has been reported for each author on the MovieReviews (Zaidan et al., 2007), SST (Socher et al., 2013), and the FEVER (Thorne et al., 2018) datasets. The predictive performance is evaluated using the F1 Score (F1), and the quality of the produced rationales is assessed using Sufficiency (Suff) and Comprehensiveness (Comp).

**Table A1:** Performance of different rationalization approaches on the MovieReviews, SST, and FEVER datasets.

**Dataset**	**Rationale**	**Approach**	**F1**	**Suff**	**Comp**	**References**
MovieReviews	Extractive	Pipeline	0.77	0.88	0.10	Atanasova et al., 2024
	Extractive	Pipeline	0.84	0.89	0.09	Guerreiro and Martins, 2021
	Extractive	Pipeline	0.91	0.95	0.12	Chan A. et al., 2022
	Extractive	MT Unsupervised	0.91	0.93	0.11	Lei et al., 2016
	Extractive	MT Unsupervised	0.94	0.92	0.12	Paranjape et al., 2020
	Extractive	MT Unsupervised	0.90	0.91	0.15	Carton et al., 2020
	Extractive	MT Supervised	0.92	0.93	0.14	Lei et al., 2016
	Extractive	MT Supervised	0.96	0.91	0.16	DeYoung et al., 2020
	Abstractive	MT Text-to-Text	0.97	0.89	0.11	Narang et al., 2020
SST	Extractive	Pipeline	0.80	0.75	0.11	Guerreiro and Martins, 2021
	Extractive	Pipeline	0.93	0.89	0.11	Chan A. et al., 2022
	Extractive	MT Unsupervised	0.92	0.95	0.15	Carton et al., 2020
	Abstractive	Generative Pipelined	0.90	0.79	0.07	Zhao and Vydiswaran, 2021
FEVER	Extractive	MT Unsupervised	0.71	0.85	0.05	DeYoung et al., 2020
	Extractive	Pipeline	0.70	0.89	0.07	Guerreiro and Martins, 2021
	Extractive	MT Unsupervised	0.82	0.85	0.15	Carton et al., 2020
	Extractive	MT Supervised	0.85	0.87	0.14	DeYoung et al., 2020
	Extractive	MT Supervised	0.87	0.87	0.16	DeYoung et al., 2020
	Abstractive	Generative MT	0.84	0.87	0.11	Zhou et al., 2020
